# Phthalates Are Metabolised by Primary Thyroid Cell Cultures but Have Limited Influence on Selected Thyroid Cell Functions *In Vitro*

**DOI:** 10.1371/journal.pone.0151192

**Published:** 2016-03-17

**Authors:** Juliana Frohnert Hansen, Marianne Møller Brorson, Malene Boas, Hanne Frederiksen, Claus Henrik Nielsen, Emma Sofie Lindström, Jacob Hofman-Bang, Marie-Louise Hartoft-Nielsen, Thomas Frisch, Katharina M. Main, Klaus Bendtzen, Åse Krogh Rasmussen, Ulla Feldt-Rasmussen

**Affiliations:** 1 Department of Medical Endocrinology, PE 2132, Rigshospitalet, University of Copenhagen, Copenhagen, Denmark; 2 Department of Growth and Reproduction, Rigshospitalet, University of Copenhagen, Copenhagen, Denmark; 3 Institute for Inflammation Research, section 7521, Rigshospitalet, University of Copenhagen, Copenhagen, Denmark; 4 Department of ENT-Head and Neck surgery, Rigshospitalet, University of Copenhagen, Copenhagen, Denmark; Hokkaido University, JAPAN

## Abstract

Phthalates are plasticisers added to a wide variety of products, resulting in measurable exposure of humans. They are suspected to disrupt the thyroid axis as epidemiological studies suggest an influence on the peripheral thyroid hormone concentration. The mechanism is still unknown as only few *in vitro* studies within this area exist. The aim of the present study was to investigate the influence of three phthalate diesters (di-ethyl phthalate, di-n-butyl phthalate (DnBP), di-(2-ethylhexyl) phthalate (DEHP)) and two monoesters (mono-n-butyl phthalate and mono-(2-ethylhexyl) phthalate (MEHP)) on the differentiated function of primary human thyroid cell cultures. Also, the kinetics of phthalate metabolism were investigated. DEHP and its monoester, MEHP, both had an inhibitory influence on 3'-5'-cyclic adenosine monophosphate secretion from the cells, and MEHP also on thyroglobulin (Tg) secretion from the cells. Results of the lactate dehydrogenase-measurements indicated that the MEHP-mediated influence was caused by cell death. No influence on gene expression of thyroid specific genes (Tg, thyroid peroxidase, sodium iodine symporter and thyroid stimulating hormone receptor) by any of the investigated diesters could be demonstrated. All phthalate diesters were metabolised to the respective monoester, however with a fall in efficiency for high concentrations of the larger diesters DnBP and DEHP. In conclusion, human thyroid cells were able to metabolise phthalates but this phthalate-exposure did not appear to substantially influence selected functions of these cells.

## Introduction

Phthalates are plasticizers used in a large variety of consumer products and building materials. They are produced as diesters but are rapidly metabolised to monoesters when entering an organism. More complex and larger phthalate molecules are further metabolised to secondary metabolites by oxidation, before they are excreted partly glucuronidated in the urine [1–3]. While the metabolism of phthalates has been previously studied in cell cultures [4–9] the kinetics of phthalate metabolism from di- to monoester are unknown.

It is well known that phthalates have anti-androgenic activity [10;11] and they are also suspected to influence the thyroid axis, reviewed in [12]. Associations between phthalate exposure and thyroid function have thus been investigated in several epidemiological studies [13–20], suggesting that phthalates may influence the concentration of peripheral thyroid hormones [13–15;18;20], though both positive and negative associations have been observed. Similarly, studies in rodents have demonstrated that phthalates may either decrease or increase peripheral thyroid hormone concentrations, with or without a concurrent change in thyroid stimulating hormone (TSH) concentrations [21–26]. One human *in vivo* study failed to detect any influence from dermal phthalate application on circulating TSH or peripheral thyroid hormone concentrations [27], as did one rodent *in vivo* study using oral phthalate exposure [28]. Few *in vitro* studies have investigated phthalate-effects on the thyroid axis and very different endpoints have been used in the study designs [29–36]. Other studies have investigated possible phthalate-mediated effects on the thyroid hormone receptor but are not relevant in relation to this study and therefore not further mentioned here.

The aim of the present study was to investigate if phthalates exerted a direct influence on human thyroid cells in primary cultures. The ability of the human thyroid cells to metabolise phthalates as well as the influence of phthalates on selected functions of these cells were investigated.

## Methods

### Cell cultures

Primary human thyroid epithelial cells were cultured as previously described [37] with minor modifications. In brief, paraadenomatous tissue removed during thyroidectomies at the Department of Ear, nose and throat (ENT)-Head and Neck surgery, Rigshospitalet, University of Copenhagen, was washed in phosphate buffered saline (PBS) (calcium and magnesium free, Gibco, Invitrogen Thermo Fischer Scientific, Waltham, MA, USA) and cut into small pieces followed by incubation with collagenase I (Sigma-Aldrich, St. Louis, MO, USA) and dispase II (Roche, Basel, Switzerland) for 75 minutes at 37°C. The digested tissue was filtered through a 100 μm filter (Falcon, BD bioscience, NJ, USA) and HAM’s F-12 culture medium supplemented with L-glutamin (Panum Institute, Copenhagen University, Denmark), 5% foetal bovine serum (FBS) (Biological Industries, Beit HaEmek, Israel), non-essential amino acids, penicillin and streptomycin (Gibco) were added. The suspension was centrifuged at 1200 x G for 5 minutes, and cells were re-suspended in HAM’s F12 culture medium containing the same supplements as mentioned above and six additional nutritional factors: TSH (1U/l, Sigma-Aldrich), insulin (Eli Lilly, Herlev, Denmark), transferrin and glycyl-histidyl-lysine acetat (Sigma-Aldrich), somatostatin and hydrocortisone (Calbiochem, EMD Millipore, Billerica, MA, USA). Cells were seeded on 24 well plates and cultured in humidified air, 37°C, 5% CO_2_, to confluent monolayers for approximately 10 days. Before initiation of the experiments, cell cultures were starved from TSH for 3 days. Experiments were conducted using culture media without FBS and in presence or absence of TSH (i.e. TSH-stimulated or unstimulated cultures). Substances to be tested (phthalates or a positive control, interleukin (IL) -1β) were added separately to experiment wells and 0.1% ethanol was added to the negative controls of phthalate experiments. Cell cultures were incubated for 24, 48 or 72 hours, before cell supernatants were harvested and stored at -20°C. The cells were harvested on ice immediately after by using lysis buffer (Qiagen, Hilden, Germany) and stored at -80°C with prior addition of 70% ethanol.

Experiments were conducted either in single determination, duplicates or triplicates, which is specified in the result section. Replicates derived from the same cell culture were grown on the same 24-well culture plate.

Primary thyroid cell cultures obtained by the described method were shown to contain 98% thyroglobulin (Tg)-producing cells [38]. The function of each thyroid cell culture was ensured by assessing the outcome variables from both TSH- and unstimulated cells on all plates. To optimise the cell cultures, the impact of TSH-starvation, addition of FBS during experiments and the duration of experiments were studied. In these studies, cell supernatants and cells were harvested as described above, except for the study of FBS-addition where no cells were harvested.

Outcome variables in the experiments were Tg and 3'-5'-cyclic adenosine monophosphate (cAMP) secretion as well as gene expression of Tg, thyroid peroxidase (TPO), sodium iodine symporter (NIS), thyroid stimulating hormone receptor (TSHr) and interleukin (IL)-6. These variables were also assessed in the presence of IL-1β (final concentrations of 100, 10^3^, 10^4^ and 10^5^ U/l and 72 h exposure; Novo Nordisk A/S, Copenhagen, Denmark). The cytokine IL-1β is known to consistently inhibit the differentiated functions of thyroid cells (reviewed in [39]) which in turn produce/secrete cytokines (such as IL-6) instead [40], and IL-1β therefore served as positive control of the experimental design.

### Phthalates

Three diesters, di-ethyl phthalate (DEP, CAS: 84-66-2), di-n-butyl phthalate (DnBP, CAS: 84-74-2) and di-(2-ethylhexyl) phthalate (DEHP, CAS: 117-81-7), and two monoesters, mono-(2-ethylhexyl) phthalate (MEHP, CAS: 4376-20-9) and mono-n-butyl phthalate (MnBP, CAS: 131-70-4) were investigated in this study. The function of the thyroid cells exposed to separate phthalates was assessed in both TSH- and unstimulated cell cultures, the latter to investigate if phthalates themselves were able to activate cells. Since pilot studies in unstimulated cultures indicated limited influence by phthalates (data not shown), only DEP, DnBP and MnBP were investigated for Tg and cAMP secretion, and DEP for gene expression in these cultures. On the other hand, TSH-stimulated cultures were exposed to all three diesters (DEHP, DnBP and DEP) and the two monoesters (MEHP and MnBP) with measurement of both Tg- and cAMP-secretion. Furthermore, the influence of the diesters (DEHP, DnBP and DEP) on gene expression was investigated.

DEP and DEHP were purchased from Aldrich Chemical Company (Sigma-Aldrich), DnBP from VWR International (Radnor, PA, USA) or Aldrich Chemical Company, MnBP from Aldrich Chemical Company or Cambridge Isotope Laboratory (Tewksbury, MA, USA) and MEHP from Cambridge Isotope Laboratory. Phthalates were dissolved in ethanol prior to further dilution in the cell culture media (final ethanol concentration was 0.1%) and added separately to the cell culture wells at a final concentration of 0.001, 0.01, 0.1, 1, 10 or 100 μM, respectively. The concentrations of mono-ethyl phthalate (MEP), MnBP and MEHP in cell culture supernatants were quantified by isotope diluted online-Turbo Flow-liquid chromatography-tandem mass spectrometry based on a method previously described [41], but modified to an 11.5 minute runtime. The metabolites were all primary metabolites of DEP, DnBP and DEHP, respectively. Inter- and intra-assay variations of cell media spiked with MEP, MnBP and MEHP, at four different concentrations showed variations from 2.9% to 8.8%. All results are given as mean ± SD.

To investigate how fast metabolism occurred, a time-study was conducted. Primary thyroid cell cultures were exposed to either DEP, DnBP or DEHP at 0.1 μM for 0, ¼, ½, 1, 2, 4, 24, 48 and 72 hours, after which cell culture supernatants were harvested and their contents of MEP, MnBP and MEHP were determined.

### cAMP

3-Isobutyl-1-methylxanthine (IBMX, Sigma-Aldrich) was added to cell cultures used for cAMP assessment concurrently with the phthalates or IL-1β. IBMX was diluted in ethanol (final ethanol concentration 1%), and thus, 1.1% ethanol was added to the ethanol-control in these experiments. Cells were harvested as described above, and the cAMP concentration was measured by a competitive protein binding method [42]. The intra-assay variation at concentrations of 0.4 and 1.4 μM was 4.7 and 7.2%, respectively (n = eight duplicates for each control level). The inter-assay variation was 13.5% for the low control (range 0.29–0.45 μM) and 9.7% for the high control (range 1.10–1.71 μM) (n = five samples in duplicates for each control). The calibration range was 0.05 to 2.0 μM.

### Thyroglobulin

Secretion of Tg was measured in supernatants by enzyme-linked immunosorbent assay (ELISA). Wells of polystyrene plates were coated with mouse Tg-antibody (Tg-Ab) (AbD Serotec, Oxford, UK). After addition of supernatants, rabbit anti-human Tg-Ab [43] was added, and plates were incubated for 60 minutes at 37°C, washed and peroxidase-conjugated polyclonal swine anti-rabbit immunoglobulin was added together with murine serum (both DAKO, Glostrup, Denmark). After 60 minutes incubation, plates were washed and a chromogenic substrate was added (TMB One, KEM EN TEC diagnostics, Taastrup, Denmark). The reaction was stopped by adding 0.18 M sulphuric acid and results were read using an ELISA reader (BioTek Synergy 2, Winooski, VT, USA) at 450 nm. The intra-assay variation at 52 and 101 μg/l was 9.5 and 8%, respectively (n = seven and six duplicates for the low and high control level, respectively). The inter-assay variation was 22.3% for the low control (range 32–65 μg/L) and 17.5% for the high control (65–121 μg/L) (n = five samples in duplicate for each control). The standard range was 10 to 500 μg/L.

### RT-qPCR

Total RNA from harvested primary human thyroid cells was extracted with an Rneasy mini kit (Qiagen) according to the manufacturer’s protocol. However, DNA elimination was not performed routinely. The concentration and purity of the achieved RNA was measured on a NanoDrop spectrophotometer (nd-1000, Wilmington, DE, USA). cDNA was synthesized (Superscript VILO synthesis kit (Invitrogen)) by mixing 4 μL of the VILO reaction mix, 2 μL of the Superscript enzyme mix, the same amount of RNA from each sample and RNAase free water to a total volume of 20 μL. Samples were incubated for 10 minutes at 25°C, 60 minutes at 50°C and 5 minutes at 85°C, after which 80 μL of 0.5X Tris-EDTA-buffer (Sigma-Aldrich) were added.

For real-time quantitative polymerase chain reaction (RT-qPCR), SYBR Green JumpStart Taq Ready Mix (Sigma-Aldrich) was used. Primers and the respective sequences are listed in S1 Table. A pool of undiluted cDNA was used for preparation of the standard curve. Four μL of SYBR Green JumpStart Taq ReadyMix, 10 μL H_2_O and 1 μL primer-mix (1μM final concentration of each primer) were added to each reaction. RT-qPCR was done on Lightcycler 480 II (Roche) with the following cycling: Initial denaturation at 94°C for 2 minutes, followed by 45 cycles of 30 seconds at 94°C and 45 seconds at 59°C and 1.30 minutes at 72°C, and final melting curve analysis. The high number of cycles was used due to very low expression of both NIS and IL-6. The specificity of the amplified products was verified by melting curve analysis and the relative mRNA quantification was achieved by standard curve method. The obtained gene of interest-expression was then divided by the housekeeping gene (HKG)-expression of the same sample.

Four different HKG were used: beta-2-microglobulin (B2M), actin, glyceraldehyde-3-phosphate dehydrogenase (GAPDH) or ATP synthase H+ transporting mitochondrial F1 complex beta polypeptide. Neither phthalates, TSH nor ethanol had an influence on these HKG. At least two HKG were included in the analysis of each experiment and the genes of interest were normalised to the most stable HKG. Stability was judged by the standard deviation of the HKG and by correlation to other HKG from the same experiment.

### Cytotoxicity

Phthalate-induced cytotoxicity was evaluated by measurement of lactate dehydrogenase (LDH) content in cell culture supernatants. A homogenous membrane integrity assay (CytoTox-ONE, Promega, Fitchburg, WI, USA) was used according to the manufacturer’s protocol with the modification that the LDH content was assessed in harvested supernatants instead of directly in the cell cultures. Briefly, one thyroid cell culture was exposed to DEHP and MEHP (0.001 to 100 μM), and both a positive Triton X-100 (0.02, 0.2 or 1.9 mg/L) control and three cell culture controls (unstimulated control, TSH-stimulated control and ethanol control) were added. Culture supernatants were harvested after 72 h of incubation and centrifuged at 1200 x G for 5 minutes. The supernatants were then transferred to a black 96-well half area microplate (Th.Geyer, Renningen, Gerrmany) followed by addition of the same volume CytoTox-One reagent to all wells. The microplate was stirred in a shaker and incubated at room temperature for 10 to 15 minutes. Hereafter, stop solution (included in the assay) was added, and the plate was again stirred before results were read on a fluorometer (Victor2, PerkinElmer, Waltham, MA, USA). The LDH contents/amount of lysed cells was proportional to the fluorescence produced and given in relative fluorescence units, as a measure of toxicity of the phthalates.

### Ethics

This study was approved by The Danish Committees on Health Research Ethics, Capital Region (Protocol number: H-1-2012-110), which in Denmark also represents the institutional review board. Oral and written informed consent was given by the study participants before donation of the thyroid tissue used for primary thyroid cell cultures.

### Statistics

Results from the method evaluation (except evaluation of incubation time) were analysed by paired T-test. For all other experiments, 2 Way ANOVA and Tukey’s Post-hoc analysis were used to compare experiment groups (SAS institute). All analyses were performed with either untransformed or log10-transformed data as appropriate (which is noted in the tables or figure legends), depending on homogeneity of variance (Bland-Altman plot) and normal distribution (histogram). If experiments were conducted in duplicates or triplicates, the mean of the outcome variable was used in analysis. P-values <0.05 were considered statistically significant.

## Results

### Thyroid cell cultures

Concentrations of cAMP and Tg in supernatants and expression of Tg-, TPO-, NIS- and TSHr-mRNA were significantly lower in unstimulated compared to TSH-stimulated thyroid cells (Table 1). TSH-starved cell cultures had higher cAMP-levels compared to non-starved cells, but only in TSH-stimulated cultures (p = 0.03, n = eight cultures in triplicates). None of the other functional variables were significantly influenced by starvation, neither in TSH- nor unstimulated cultures (S2 Table).

**Table 1:** Levels of outcome variables in un- and TSH-stimulated controls. Experiments were conducted in duplicates or triplicates and exposure time was 72 h. Log10-transformed data was used in statistical analysis. cAMP: 3'-5'-cyclic adenosine monophosphate. IL: interleukin. NIS: sodium iodine symporter. Tg: thyroglobulin. TPO: thyroid peroxidase. TSHr: thyroid stimulating hormone receptor.

Outcome variables	n	unstimulated control	TSH-stimulated control	unit	P-value
**cAMP**	23	0.00002 (0–0.15)	0.48 (0.06–4.04)	μM	<0.0001
**Tg**	22	556 (76–72,052)	1020 (104–83,649)	μg/L	0.0003
**Tg mRNA**	17	0.2 (0.01–1.2)	0.7 (0.01–3.5)	ratio to housekeeping gene	0.0008
**TPO mRNA**	18	0.2 (0.04–1.1)	1.2 (0.1–4.4)	ratio to housekeeping gene	<0.0001
**NIS mRNA**	16	0.04 (0–2.9)	1.5 (0.4–72)	ratio to housekeeping gene	0.0001
**TSHr mRNA**	18	0.8 (0.2–2.3)	1.0 (0.08–3.4)	ratio to housekeeping gene	0.04

Absence of FBS during experiments resulted in higher cAMP levels and unchanged Tg levels compared to experiments conducted in presence of FBS (p = 0.04, three cultures in triplicates), yet, only observed in TSH-stimulated cultures (S2 Table).

For evaluation of incubation time, cells and supernatants were harvested after 6, 12, 48 or 72 hours, respectively. All assessed variables increased over time. Thus, the Tg-secretion was higher after 72 hours than at all other time points, whereas cAMP-secretion stagnated after 48 hours (S3 Table). Gene expression of all assessed genes was higher after 24, 48 or 72 hours compared to 6 hours. However, there was no significant difference between these three time points (24, 48 and 72 hours), except for Tg- and TPO- mRNA where expression was higher after 48 and 72 hours, respectively, compared to 24 hours (S3 Table).

The positive control cell cultures exposed to IL-1β for 72 hours in TSH-stimulated cultures demonstrated a dose-dependent inhibition of both Tg and cAMP secretion by this cytokine. Likewise, the expression of all thyroid specific genes was impaired in a dose dependent manner, while gene expression of the cytokine IL-6 was enhanced by IL-1β (S1 Fig).

### Metabolism of phthalates

The monoesters MnBP and MEHP, when added to cell cultures, were fully recovered at all added concentrations. All three diesters, DEP, DnBP and DEHP, were metabolised to their respective primary monoester, MEP, MnBP and MEHP. No secondary metabolites of DEHP, such as mono-(2-ethyl-5-hydroxyhexyl) phthalate, mono-(2-ethyl-5-oxohexyl) phthalate or mono-(2-ethyl-5-carboxypentyl) phthalate were detected.

**Fig 1:**
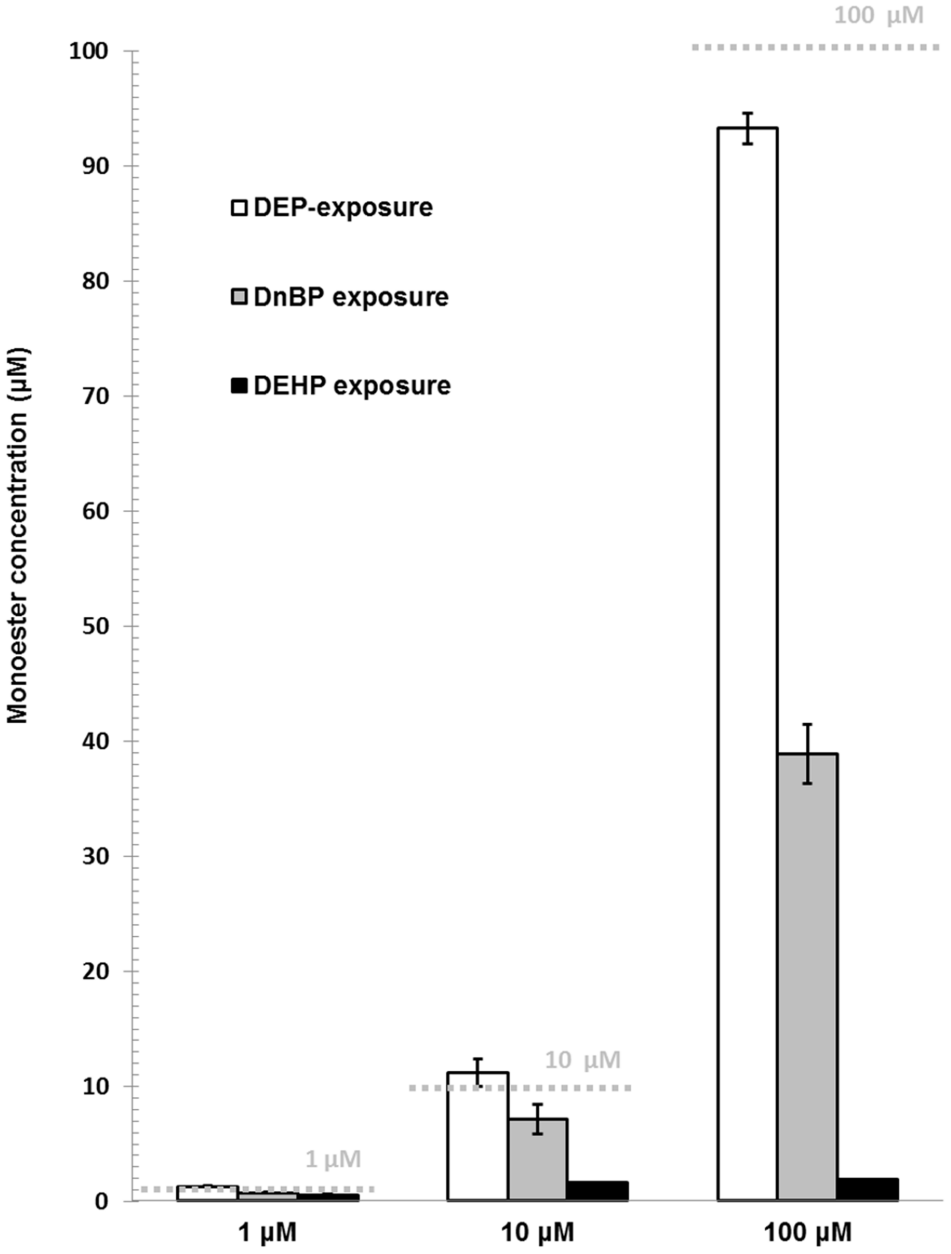
Monoester concentrations (mean ± SD) in cell culture supernatants with the highest added diester-concentrations (1 to 100 μM). N = DEP: one culture in duplicates; DnBP and DEHP: two cultures in single determination except for 100 μM DEHP, with only one culture in single determination due to removal of a single outlier. Grey dashed lines indicate the expected monoester concentration if all diester had been metabolised. DEP: di-ethyl phthalate. DnBP: di-n-butyl phthalate. DEHP: di-2-ethylhexyl phthalate.

**Fig 2:**
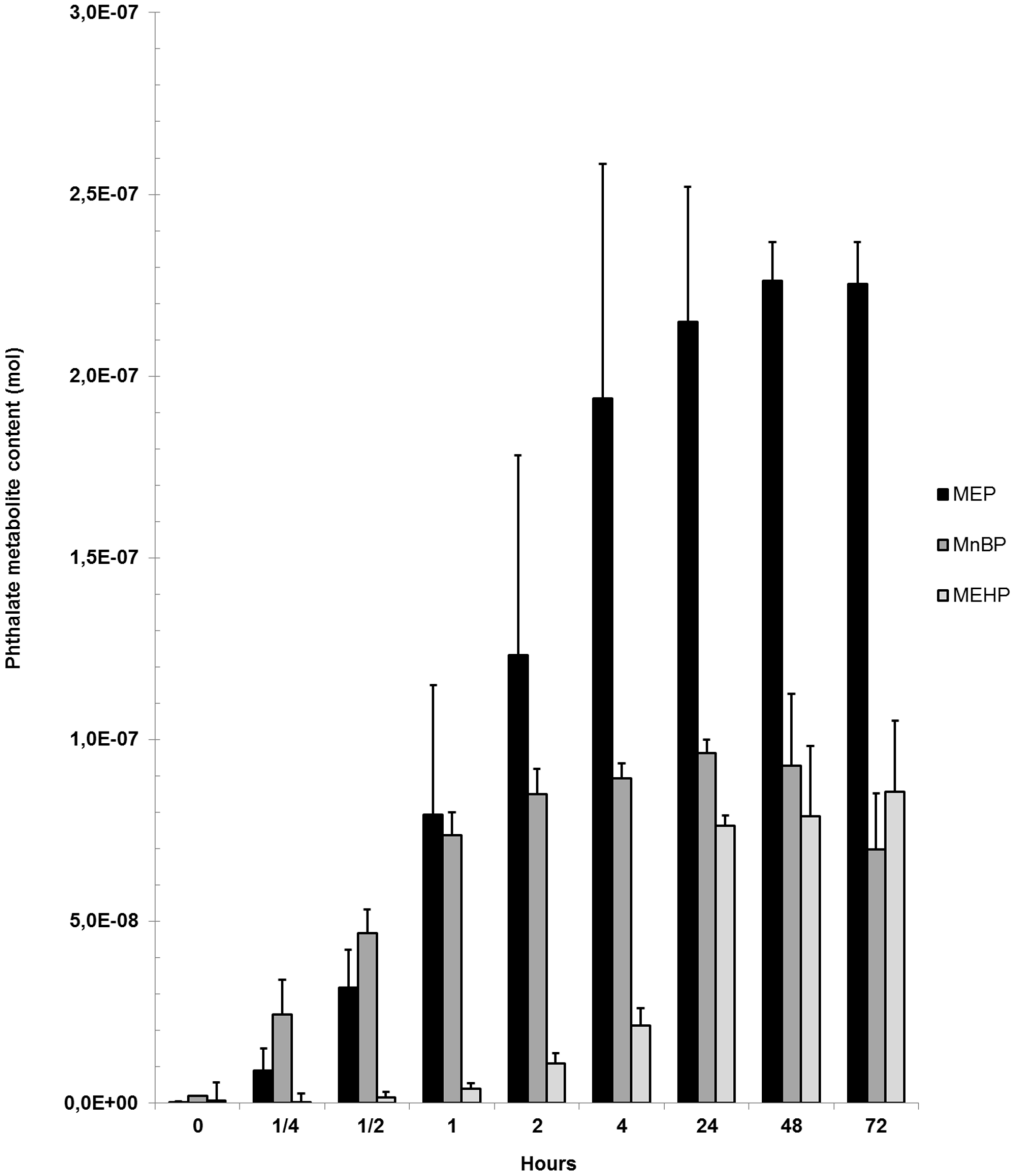
Time study of phthalate diester metabolism. The measured content of MEP, MnBP and MEHP are expressed as mean ± SD at time points 0 to 72 hours after phthalate diester addition (0.1 μM DEP, DnBP or DEHP) to TSH-stimulated cell cultures (n = three cultures in single determination). The monoester concentrations are corrected for background contamination. MEP: mono-ethyl phthalate. MnBP: mono-n-butyl phthalate. MEHP: mono-2-ethylhexyl phthalate.

Cell cultures exposed to the low diester-concentrations (0.001 up to 0.1 μM) had higher measurable monoester concentrations than the added diester-concentration. Thus, for instance 0.3 μM MnBP was measured in cell cultures exposed to 0.1 μM DnBP (data not shown). For the high diester-concentrations (1 to 100 μM), DEP was almost fully metabolised to the monoester MEP while metabolism of DnBP and DEHP to their respective monoester declined with increasing concentrations of the diester (Fig 1). There was a time-dependent increase in the monoester content of all three added diesters (0.1 μM). The highest contents of MnBP, MEP and MEHP were found after 24, 48 and 72 hours, respectively. After 4 hours, 86%, 93% and 25% of the respective maximal content of MEP, MnBP and MEHP were observed (Fig 2).

### Cell function of phthalate-exposed cells

**Fig 3:**
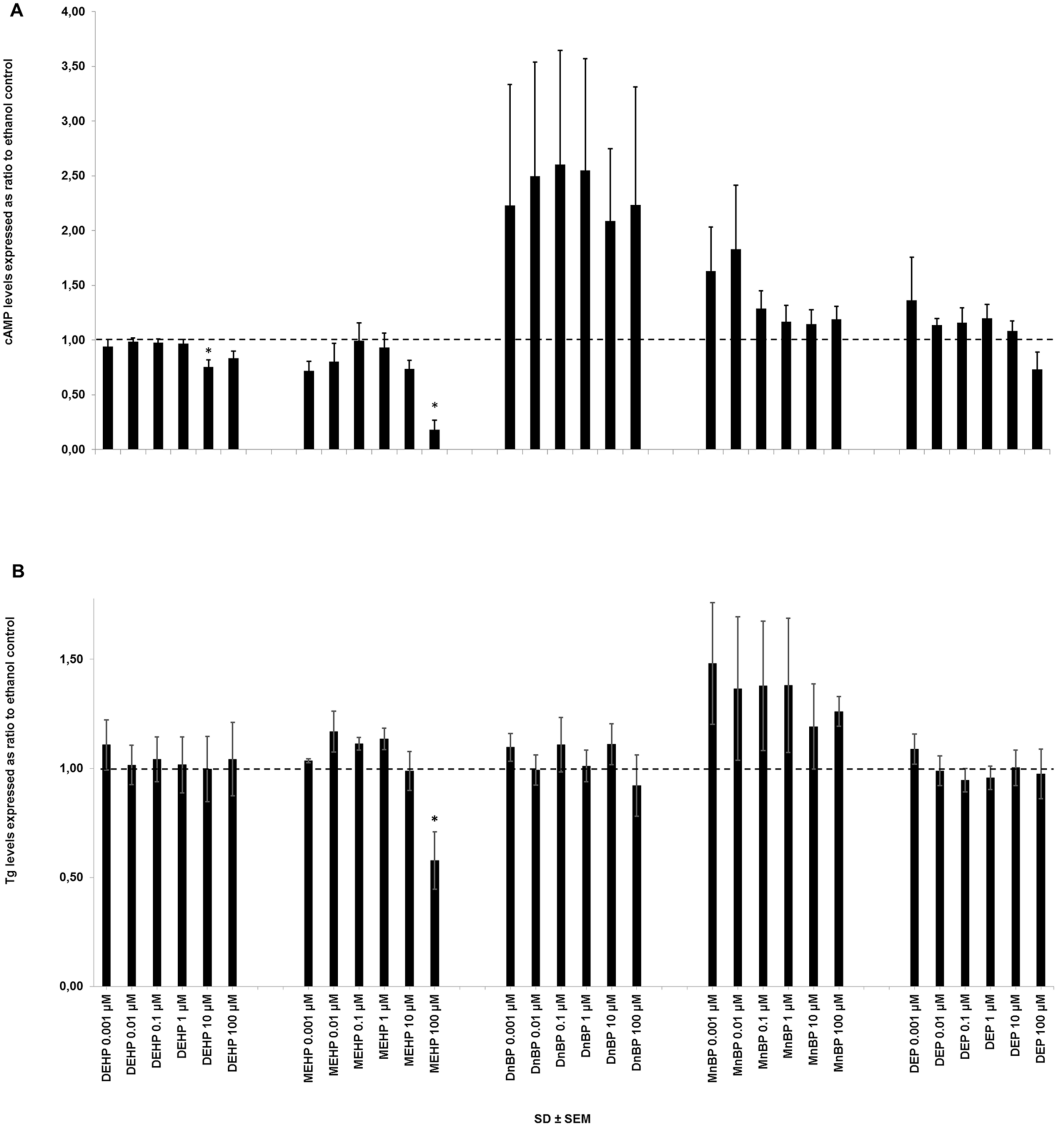
cAMP- (A) and Tg- (B) secretion from TSH-stimulated primary human thyroid cells. Cells were exposed to DEHP (n = 9 cultures in single determination), MEHP (n = 3 cultures in single determination), DnBP (n = 8 (A) or 10 (B) cultures in single determination or duplicates), MnBP (n = 6 (A) or 10 (B) cultures in single determination) or DEP (n = 6 (A) or 11 (B) cultures in single determination or duplicates) in six different concentrations (0.001 to 100 μM) for 72 h. cAMP- and Tg-concentrations are expressed as ratio to the respective ethanol control. The dashed line indicates ratio = 1 (control levels). The measured median and range (parenthesis) of the ethanol controls was: 1.3 (0.2–4.4) μM) for cAMP and 26,202 (176–87,923) μg/L) for Tg. * p<0.05 in ANOVA. cAMP: 3'-5'-cyclic adenosine monophosphate. DEHP: di-2-ethylhexyl phthalate. DEP: di-ethyl phthalate. DnBP: di-n-butyl phthalate. MEHP: mono-2-ethylhexyl phthalate. MnBP: mono-n-butyl phthalate. Tg: thyroglobulin. TSH: thyroid stimulation hormone.

After 72 hours of incubation in TSH-stimulated cultures, both DEHP and MEHP had a significant influence on cAMP-secretion (DEHP: p = 0.0006, n = nine cultures in single determination; MEHP p = 0.01, n = three cultures in single determination) (Fig 3A). Comparison of exposure groups by post-hoc analysis demonstrated an inhibition by 10 μM DEHP compared to the control, 1, 0.1 and 0.01 μM, and an inhibition by 100 μM MEHP compared to all other concentrations (S4 Table). DnBP seemed to stimulate cAMP-secretion, which however was not statistically significant (Fig 3A and S4 Table). Studying the single courses of these experiments revealed the following: in three out of eight experiments, DnBP stimulated cAMP-secretion, which was very marked for one culture; in the remaining other five cultures the cAMP-secretion was either below or about the same levels as that of the ethanol control (S2 Fig).

None of the investigated phthalates had any influence on cAMP-secretion in unstimulated cultures (S4 Table).

Only MEHP had a significant influence on Tg-secretion from TSH-stimulated cultures after 72 hours of incubation (p = 0.01, n = three cultures in single determination) (Fig 3B). Comparison of exposure groups by post-hoc analysis demonstrated an inhibition by 100 μM MEHP compared to all other concentrations. None of the investigated phthalates had any influence on Tg-secretion in unstimulated cultures (S4 Table).

Gene expression of all investigated genes (Tg, TPO, NIS, TSHr and IL-6) in TSH- and unstimulated cultures was not significantly influenced by any of the investigated phthalates (72 h phthalate-exposure) (S4 Table).

A time study (using incubation times of 24, 48 or 72 h) showed no significant influence on cAMP- or Tg-secretion nor on Tg-mRNA-expression in cell cultures exposed to concentrations from 0.001 to 100 μM DEHP (data not shown).

### Cytotoxicity

The LDH-content of the three negative culture controls (TSH-stimulated, unstimulated and ethanol control) was lower than the positive Triton X-100 control at the two highest concentrations (0.2 and 1.9 mg/L). Supernatants of cell cultures exposed to MEHP at 100 μM had a higher LDH-content compared to all negative culture controls. At all other concentrations, MEHP and DEHP resulted in an LDH-content lower than or similar to the negative culture controls (S3 Fig).

## Discussion

In the present *in vitro* study, a limited influence on the function of primary thyroid cell cultures by all the investigated phthalate di- and monoesters was found. Experiments with phthalates were mainly conducted in single determination in order to accommodate all dilutions and controls for comparison on the same culture plate, while the number of experiments was sought to be at least nine. Since no major dose-response effects were detectable, especially compared to the positive control IL-1β, some phthalates and outcomes were studied in less than nine experiments. Thus results are only indicative, but still conclusive in that no major or dose-dependent influences were found.

Only DEHP at the second highest concentration (10 μM) inhibited cAMP-secretion. MEHP at the highest concentration (100 μM) inhibited both cAMP- and Tg-secretion but also resulted in an increased LDH-release. Thus, the observed influences at this high concentration were probably caused by cell death resulting in less production of cAMP and Tg, rather than mere modulation of the cellular function. Also, a high ethanol concentration (1.1%) was present in cultures where cAMP was measured and could itself act in synergy with the phthalates and result in a toxic influence. Only one other *in vitro* study has investigated the influence on cAMP production (human recombinant (hr)-TSHr-mediated) by phthalic acid where no significant influence was described [34].

Gene-expression of Tg, NIS, TPO and TSHr, which increased with culture time and were therefore chosen to be assessed after 72 h of incubation, were unaffected by DEP, DnBP or DEHP-exposure in the present study. A recent *in vivo* study by Liu et al. of Sprague-Dawley rats demonstrated a decrease in serum TPO, NIS and peripheral thyroid hormones, but unchanged concentrations of serum Tg, TSH and thyrotropin releasing hormone by DEHP [44]. The group of Ulrich Loos investigated how phthalates influenced the iodine uptake and NIS gene expression in rodent cell lines (FRTL-5 and PC C13, respectively) [29;36]. DEHP but not di-butyl phthalate (DBP) increased iodine uptake, while neither DEHP nor DBP affected NIS mRNA expression, which on the other hand was influenced by other investigated phthalates [29;36]. Song et al. investigated the activity of the hrTPO gene and found it to be increased by DBP (100 μM) [35]. In summary, no other or only few published studies have investigated phthalate mediated influences on Tg, NIS, TPO or TSHr. Of these studies, four (including the present study) have investigated NIS. Thus, DEHP did not seem to affect the gene expression of NIS, but may influence iodine uptake. NIS in serum, which was analysed by a commercial ELISA-kit [44], could also derive from other cells than thyroid cells [45], making assumptions on phthalate mediated influence on the thyroid-derived NIS uncertain.

To confirm that our cell system was suitable for investigation of inhibiting substances, IL-1β, was used as a positive control since its influence on the release of Tg and cAMP from primary and secondary human thyroid cell cultures has repeatedly and reproducibly been demonstrated [37;40;46–48]. In the present study, a similar influence on thyroid specific gene expression level was seen. Thus, IL-1β caused a dose-dependent decrease in thyroid specific genes (TPO, TSHr, NIS and Tg) but increased the IL-6 gene expression [40]. Only one experiment with IL-1β was conducted in the present study and judged sufficient since results confirmed those of numerous previous studies with IL-1β in our laboratory [37;40;46–48].

Phthalate-metabolism has previously been studied in cell cultures [4–9]. Kristensen et al. demonstrated an uptake of phthalate diesters but not monoesters by SC5 cells (juvenile mouse Sertoli cell line) and consequent metabolism of the diesters to the respective monoesters [4]. Phthalate metabolites of the present study were assessed in supernatants and thus, no firm assumption can be made about the cellular uptake of the two monoesters, MnBP and MEHP, by primary thyroid cells. However, when diesters were added to thyroid cells, the monoester concentration was increased dose-dependently, suggesting that the diesters were taken up and metabolised by the primary thyroid cell cultures. The extent and timing of metabolism differed for the three diesters: DEP was metabolised at all concentrations, while DnBP and DEHP were metabolised less effectively with increasing concentrations. DnBP and DEHP are less water-soluble than DEP and may not have been dissolved properly at the high concentrations, reducing their availability to the cells. Another observation was that DEP and DnBP appeared to be metabolised faster than DEHP, which could be due to different mechanisms of uptake into the cells due to the larger molecular size of DEHP.

FBS contains proteins, immunoglobulins and enzymes, and could therefore influence the assays used for both Tg and cAMP-quantification and also the metabolism of phthalates. FBS was not present during the experiments but other substances present in the culture media during the experiments could potentially influence the metabolism of phthalates. Phenol red has for instance oestrogen-like activity [49] and oestrogen receptors are also present in normal thyroid tissue [50]. Thus, phenol red may influence the function of thyroid cell cultures and thereby their ability to metabolise phthalates, which needs further investigation.

The phthalate concentration range used in this study included doses likely to be found in humans. Urinary concentrations of MEP in healthy Danish adolescents can reach levels as high as 13 mg/l (67 μM) [51]. In high-exposed groups, such as preterm children, the MnBP concentration in urine reached as high as 6.5 mg/l (29 μM) [52]. Since no previous study has used primary human thyroid cultures, and thus no data exist on the potency of phthalates on the function of these cells, we decided to study single phthalates in a broad concentration range instead of phthalate mixtures, which eventually may be a more realistic *in vivo* scenario. When phthalates were added to cell cultures at low concentrations (0.001, 0.01 or 0.1 μM), the measured monoester concentrations were higher than expected. In previous studies performed in our laboratory, both high background contamination of some monoesters (probably through phthalate containing laboratory equipment), and migration of some phthalates to adjacent wells were found [53]. One or both of these observations may account for the unexpected high monoester concentrations found in the present study. Thus, there is a risk that the observed influences on Tg and cAMP secretion in this study may have been caused by a mixture instead of single phthalates, since this could not be corrected for in the statistical analyses.

The purity and correct function of thyroid cells in the cultures have previously been investigated by measuring both the content of Tg-producing cells [38], and the responsiveness to TSH resulting in increased cAMP and Tg secretion, since TSH is the specific stimulator of thyroid cells both *in vivo* and *in vitro* [54]. The hormonal end products, triiodothyronine and thyroxine were previously shown to be released from the human thyroid cultures in very small quantities, probably due to absence of iodine in the culture media (own unpublished data). In the present study, the secretion of cAMP, Tg as well as gene-expression of thyroid specific genes (Tg, TPO, NIS and TSHr) were increased in TSH-stimulated cell cultures, confirming their differentiated thyroid cell functions. The 2% non-Tg producing cells present in primary thyroid cell cultures could theoretically be immune cells. As we also assessed the cell cultures gene expression of IL-6, this cytokine mRNA could theoretically originate from these immune cells, but not very likely considering the concentrations.

A limitation of studies with primary thyroid cell cultures in monolayer is their structural difference compared to thyroid cells arranged as follicles *in vivo*. Monolayers have a different cellular polarity and may also differ in the connections in-between cells compared to *in vivo* follicles. Consequently, phthalates may influence thyroid cells differently *in vivo* compared to the *in vitro* monolayers.

## Conclusion

Human thyroid cell cultures were able to metabolise phthalates from diesters to the respective monoesters. Most of the metabolism occurred during the first 24 hours and the cells metabolised DnBP and DEP faster than DEHP. A less effective metabolism of high concentrations of DnBP and DEHP was also observed. Only DEHP at 10 μM and MEHP at 100 μM were able to influence Tg- and/or cAMP-secretion. Though only investigated for DEHP, gene expression of thyroid specific genes were unaffected by DEHP exposure. For MEHP, the findings were probably caused by toxicity indicated by an increase in the supernatant-LDH-content. Though some experiments were conducted in low numbers and results thus are only indicative, no sign of major non-toxic or dose-dependent influence were seen in our studies. Proposed hypotheses from epidemiological studies of a direct effect of phthalates on the thyroid gland were not substantiated by the present *in vitro* study of differentiated gene expression as well as cAMP and protein synthesis from cultures human thyroid cells. An effect of the phthalates on thyroid hormone secretion is therefore unlikely, and such possible effects should therefore be looked for in other parts of the thyroid axis [55].

## Supporting Information

S1 Fig Function of TSH-stimulated human thyroid cell cultures exposed to IL-1β. Cell cultures were exposed to IL-1β (100 to 10^5^ U/l) for 72 hours (n = one culture in single determination). The dashed line indicates the level of the ethanol controls (ratio = 1). Tg- and cAMP-secretion was assessed in supernatants and mRNA in cells. cAMP: 3'-5'-cyclic adenosine monophosphate. Tg: thyroglobulin. TPO: thyroid peroxidase. NIS: sodium iodine symporter. TSHr: thyroid stimulating hormone receptor. IL: interleukin.
(TIF)

S2 Fig cAMP secretion from individual cell cultures (culture 1–7) exposed to DnBP (0.001 to 100 μM) for 72 hours. The dashed line indicates the level of the ethanol controls (ratio = 1). DnBP: di-n-butyl phthalate.
(TIF)

S3 Fig LDH-contents (y-axis) in supernatants from TSH-stimulated thyroid cell cultures. Cells were exposed to DEHP and MEHP, as well as positive cytotoxicity- (i.e. cell cultures exposed to Triton X-100) and negative culture- (i.e. cell cultures not exposed to phthalates) controls for 72 h. N = one culture in single determination except for the negative controls. The LDH-content was proportional to the produced fluorescence (given in relative fluorescence units (RFU)). DEHP: di-2-ethylhexyl phthalate. MEHP: mono-2-ethylhexyl phthalate.
(TIF)

S1 Table Sequences of the primers used in RT-qPCR. (PDF)

S2 Table The impact of TSH-starvation (A) and addition of FBS (B) during experiments. Results of the outcome variables were analysed by paired T-test and experiment duration was 72 h. A: outcome from experiments with thyroid stimulation hormone (TSH) -starvation are compared to those without TSH-starvation. B: outcome from experiments without foetal bovine serum (FBS) are compared to those with FBS. Experiments were conducted in triplicates. cAMP: 3'-5'-cyclic adenosine monophosphate. IL: interleukin. NIS: sodium iodine symporter. Tg: thyroglobulin. TPO: thyroid peroxidase. TSHr: thyroid stimulating hormone receptor.
(PDF)

S3 Table ANOVA and Tukey post-hoc results from the study of the culture duration. TSH-stimulated primary thyroid cells were cultured for 6, 24, 48 or 72 h, before supernatants and cells were harvested and outcome variables were analysed. All experiments were conducted in triplicates. cAMP: 3'-5'-cyclic adenosine monophosphate. IL: interleukin. NIS: sodium iodine symporter. Tg: thyroglobulin. TPO: thyroid peroxidase. TSHr: thyroid stimulating hormone receptor.
(PDF)

S4 Table Overview of 2 way ANOVA and post-hoc Tukey results from phthalate-exposed (72 h) thyroid cell cultures, in TSH- and unstimulated controls. If log10 of data was used, the estimated differences between groups are expressed as ratios, e.g. the mean cAMP-secretion from 10 μM DEHP-exposed cells was 21% lower than the mean cAMP-secretion from 0.001 μM DEHP-exposed cells, and lies with 95% certainty between 37% below and 0% below the mean of 0.001 μM DEHP-exposed cells. The hyphen (-) indicates that no post-hoc analysis was made due to insignificant 2 way ANOVA results. cAMP: 3'-5'-cyclic adenosine monophosphate. DEHP: di-2-ethylhexyl phthalate. DEP: di-ethyl phthalate. DnBP: di-n-butyl phthalate. MEHP: mono-2-ethylhexyl phthalate. MnBP: mono-n-butyl phthalate. IL: interleukin. NIS: sodium iodine symporter. Tg: thyroglobulin. TPO: thyroidperoxidase. TSH: thyroid stimulating hormone. TSHr: thyroid stimulating hormone receptor.
(PDF)

## References

[1] Frederiksen H, Skakkebaek NE, Andersson AM. Metabolism of phthalates in humans. Mol Nutr Food Res 2007 7;51(7):899–911. 17604388 10.1002/mnfr.200600243

[2] Koch HM, Bolt HM, Angerer J. Di(2-ethylhexyl)phthalate (DEHP) metabolites in human urine and serum after a single oral dose of deuterium-labelled DEHP. Arch Toxicol 2004 3;78(3):123–30. 14576974 10.1007/s00204-003-0522-3

[3] Silva MJ, Barr DB, Reidy JA, Kato K, Malek NA, Hodge CC, et al Glucuronidation patterns of common urinary and serum monoester phthalate metabolites. Archives of Toxicology 2003 10;77(10):561–7. 14574443 10.1007/s00204-003-0486-3

[4] Kristensen DM, Skalkam ML, Audouze K, Lesne L, Desdoits-Lethimonier C, Frederiksen H, et al Many putative endocrine disruptors inhibit prostaglandin synthesis. Environ Health Perspect 2011 4;119(4):534–41. 10.1289/ehp.1002635 21081300 PMC3080937

[5] Lhuguenot J-C, Mitchell AM, Milner G. The metabolism of di(2-ethylhexyl) phthalate (DEHP) and mono(2-ethylhexyl) phthalate (MEHP) in rats: In vivo and in vitro dose and time dependency of metabolism. Toxicology and Applied Pharmacology 1985;80(1):1985. 10.1016/0041-008x(85)90096-1 4024100

[6] Desdoits-Lethimonier C, Albert O, Le BB, Perdu E, Zalko D, Courant F, et al Human testis steroidogenesis is inhibited by phthalates. Hum Reprod 2012 5;27(5):1451–9. 10.1093/humrep/des069 22402212

[7] Albro PW, Chapin RE, Corbett JT, Schroeder J, Phelps JL. Mono-2-ethylhexyl phthalate, a metabolite of di-(2-ethylhexyl) phthalate, causally linked to testicular atrophy in rats. Toxicol Appl Pharmacol 1989 9 1;100(2):193–200. 2781553 10.1016/0041-008x(89)90305-0

[8] Muczynski V, Cravedi JP, Lehraiki A, Levacher C, Moison D, Lecureuil C, et al Effect of mono-(2-ethylhexyl) phthalate on human and mouse fetal testis: In vitro and in vivo approaches. Toxicol Appl Pharmacol 2012 5 15;261(1):97–104. 10.1016/j.taap.2012.03.016 22484159

[9] Sandermann H Jr, Scheel D, vdTrenck T. Use of plant cell cultures to study the metabolism of environmental chemicals. Ecotoxicol Environ Saf 1984 4;8(2):167–82. 6325118 10.1016/0147-6513(84)90059-9

[10] Kay VR, Bloom MS, Foster WG. Reproductive and developmental effects of phthalate diesters in males. Crit Rev Toxicol 2014 7;44(6):467–98. 10.3109/10408444.2013.875983 24903855

[11] Skakkebaek NE, Rajpert-De ME, Main KM. Testicular dysgenesis syndrome: an increasingly common developmental disorder with environmental aspects. Hum Reprod 2001 5;16(5):972–8. 11331648 10.1093/humrep/16.5.972

[12] Boas M, Feldt-Rasmussen U, Main KM. Thyroid effects of endocrine disrupting chemicals. Mol Cell Endocrinol 2012 5 22;355(2):240–8. 10.1016/j.mce.2011.09.005 21939731

[13] Boas M, Frederiksen H, Feldt-Rasmussen U, Skakkebaek NE, Hegedus L, Hilsted L, et al Childhood exposure to phthalates: associations with thyroid function, insulin-like growth factor I, and growth. Environ Health Perspect 2010 10;118(10):1458–64. 10.1289/ehp.0901331 20621847 PMC2957929

[14] Meeker JD, Calafat AM, Hauser R. Di(2-ethylhexyl) phthalate metabolites may alter thyroid hormone levels in men. Environ Health Perspect 2007 7;115(7):1029–34. 17637918 10.1289/ehp.9852 PMC1913587

[15] Meeker JD, Ferguson KK. Relationship between urinary phthalate and bisphenol A concentrations and serum thyroid measures in U.S. adults and adolescents from the National Health and Nutrition Examination Survey (NHANES) 2007–2008. Environ Health Perspect 2011 10;119(10):1396–402. 10.1289/ehp.1103582 21749963 PMC3230451

[16] Rais-Bahrami K, Nunez S, Revenis ME, Luban NL, Short BL. Follow-up study of adolescents exposed to di(2-ethylhexyl) phthalate (DEHP) as neonates on extracorporeal membrane oxygenation (ECMO) support. Environ Health Perspect 2004 9;112(13):1339–40. 15345350 10.1289/ehp.6901 PMC1247527

[17] Wang Q, Wang L, Chen X, Rao KM, Lu SY, Ma ST, et al Increased urinary 8-hydroxy-2'-deoxyguanosine levels in workers exposed to di-(2-ethylhexyl) phthalate in a waste plastic recycling site in China. Environ Sci Pollut Res Int 2011 7;18(6):987–96. 10.1007/s11356-010-0420-1 21298484

[18] Huang PC, Kuo PL, Guo YL, Liao PC, Lee CC. Associations between urinary phthalate monoesters and thyroid hormones in pregnant women. Hum Reprod 2007 10;22(10):2715–22. 17704099 10.1093/humrep/dem205

[19] de Cock M, de Boer MR, Lamoree M, Legler J, van de Bor M. Prenatal exposure to endocrine disrupting chemicals in relation to thyroid hormone levels in infants—a Dutch prospective cohort study. Environ Health 2014;13:106 10.1186/1476-069X-13-106 25495114 PMC4293007

[20] Brucker-Davis F, Ferrari P, Boda-Buccino M, Wagner-Mahler K, Pacini P, Gal J, et al Cord blood thyroid tests in boys born with and without cryptorchidism: correlations with birth parameters and in utero xenobiotics exposure. Thyroid 2011 10;21(10):1133–41. 10.1089/thy.2010.0459 21875366

[21] Gayathri NS, Dhanya CR, Indu AR, Kurup PA. Changes in some hormones by low doses of di (2-ethyl hexyl) phthalate (DEHP), a commonly used plasticizer in PVC blood storage bags & medical tubing. Indian J Med Res 2004 4;119(4):139–44. 15147118

[22] Elcombe CR, Odum J, Foster JR, Stone S, Hasmall S, Soames AR, et al Prediction of rodent nongenotoxic carcinogenesis: evaluation of biochemical and tissue changes in rodents following exposure to nine nongenotoxic NTP carcinogens. Environ Health Perspect 2002 4;110(4):363–75. 11940454 10.1289/ehp.02110363 PMC1240799

[23] Hinton RH, Mitchell FE, Mann A, Chescoe D, Price SC, Nunn A, et al Effects of phthalic acid esters on the liver and thyroid. Environ Health Perspect 1986 12;70:195–210. 3830106 10.1289/ehp.8670195 PMC1474287

[24] O'Connor JC, Frame SR, Ladics GS. Evaluation of a 15-day screening assay using intact male rats for identifying antiandrogens. Toxicological Sciences 2002 9;69(1):92–108. 12215663 10.1093/toxsci/69.1.92

[25] Tonk EC, Verhoef A, Gremmer ER, van Loveren H, Piersma AH. Relative sensitivity of developmental and immune parameters in juvenile versus adult male rats after exposure to di(2-ethylhexyl) phthalate. Toxicol Appl Pharmacol 2012 4 1;260(1):48–57. 10.1016/j.taap.2012.01.018 22310177

[26] Dhanya CR, Gayathri NS, Mithra K, Nair KV, Kurup PA. Vitamin E prevents deleterious effects of di (2-ethyl hexyl) phthalate, a plasticizer used in PVC blood storage bags. Indian J Exp Biol 2004 9;42(9):871–5. 15462179

[27] Janjua NR, Mortensen GK, Andersson A-M, Kongshoj B, Skakkebaek NE, Wulf HC. Systemic uptake of diethyl phthalate, dibutyl phthalate, and butyl paraben following whole-body topical application and reproductive and thyroid hormone levels in humans. Environmental Science and Technology 2007 8 1;41(15):5564–70. 17822133 10.1021/es0628755

[28] Bernal CA, Martinelli MI, Mocchiutti NO. Effect of the dietary exposure of rat to di(2-ethyl hexyl) phthalate on their metabolic efficiency. Food Addit Contam 2002 11;19(11):1091–6. 12456281 10.1080/02652030210157709

[29] Breous E, Wenzel A, Loos U. The promoter of the human sodium/iodide symporter responds to certain phthalate plasticisers. Mol Cell Endocrinol 2005 12 1;244(1–2):75–8. 16257484 10.1016/j.mce.2005.06.009

[30] Shen O, Du G, Sun H, Wu W, Jiang Y, Song L, et al Comparison of in vitro hormone activities of selected phthalates using reporter gene assays. Toxicol Lett 2009 12 1;191(1):9–14. 10.1016/j.toxlet.2009.07.019 19643168

[31] Ghisari M, Bonefeld-Jorgensen EC. Effects of plasticizers and their mixtures on estrogen receptor and thyroid hormone functions. Toxicol Lett 2009 8 25;189(1):67–77. 10.1016/j.toxlet.2009.05.004 19463926

[32] Ishihara A, Sawatsubashi S, Yamauchi K. Endocrine disrupting chemicals: interference of thyroid hormone binding to transthyretins and to thyroid hormone receptors. Mol Cell Endocrinol 2003 1 31;199(1–2):105–17. 12581883 10.1016/s0303-7207(02)00302-7

[33] Ishihara A, Nishiyama N, Sugiyama S, Yamauchi K. The effect of endocrine disrupting chemicals on thyroid hormone binding to Japanese quail transthyretin and thyroid hormone receptor. Gen Comp Endocrinol 2003 10 15;134(1):36–43. 13129501 10.1016/s0016-6480(03)00197-7

[34] Santini F, Vitti P, Ceccarini G, Mammoli C, Rosellini V, Pelosini C, et al In vitro assay of thyroid disruptors affecting TSH-stimulated adenylate cyclase activity. J Endocrinol Invest 2003 10;26(10):950–5. 14759065 10.1007/BF03348190

[35] Song M, Kim YJ, Song MK, Choi HS, Park YK, Ryu JC. Identification of classifiers for increase or decrease of thyroid peroxidase activity in the FTC-238/hTPO recombinant cell line. Environ Sci Technol 2011 9 15;45(18):7906–14. 10.1021/es200475k 21809831

[36] Wenzel A, Franz C, Breous E, Loos U. Modulation of iodide uptake by dialkyl phthalate plasticisers in FRTL-5 rat thyroid follicular cells. Mol Cell Endocrinol 2005 12 1;244(1–2):63–71. 16289305 10.1016/j.mce.2005.02.008

[37] Krogh Rasmussen A, Bech K, Feldt-Rasmussen U, Poulsen S, Holten I, Ryberg M, et al Interleukin-1 affects the function of cultured human thyroid cells. Allergy 1988 8;43(6):435–41. 2847577 10.1111/j.1398-9995.1988.tb00915.x

[38] Rasmussen AK, Kayser L, Perrild H, Brandt M, Bech K, Feldt-Rasmussen U. Human thyroid epithelial cells cultured in monolayers. I. Decreased thyroglobulin and cAMP response to TSH in 12-week-old secondary and tertiary cultures. Mol Cell Endocrinol 1996 2 5;116(2):165–72. 8647316 10.1016/0303-7207(95)03711-x

[39] Rasmussen AK, Bendtzen K, Feldt-Rasmussen U. Thyrocyte-interleukin-1 interactions. Exp Clin Endocrinol Diabetes 2000;108(2):67–71. 10826510 10.1055/s-2000-5797

[40] Diamant M, Kayser L, Rasmussen AK, Bech K, Feldt-Rassmussen U. Interleukin-6 production by thyroid epithelial cells. Enhancement by interleukin-1. Autoimmunity 1991;11(1):21–6. 1812993 10.3109/08916939108994704

[41] Frederiksen H, Jorgensen N, Andersson AM. Correlations between phthalate metabolites in urine, serum, and seminal plasma from young Danish men determined by isotope dilution liquid chromatography tandem mass spectrometry. J Anal Toxicol 2010 9;34(7):400–10. 20822678 10.1093/jat/34.7.400

[42] Madsen SN, Badawi I, Skovsted L. A simple competitive protein-binding assay for adenosine-3',5'-monophosphate in plasma and urine. Acta Endocrinol (Copenh) 1976 1;81(1):208–14. 174364 10.1530/acta.0.0810208

[43] Feldt-Rasmussen U. Purification of human thyroglobulin for radioimmunoassay and testing by ultracentrifugal analysis and immunoelectrophoresis. J Immunol Methods 1978;21(3–4):295–303. 670715 10.1016/0022-1759(78)90156-4

[44] Liu C, Zhao L, Wei L, Li L. DEHP reduces thyroid hormones via interacting with hormone synthesis-related proteins, deiodinases, transthyretin, receptors, and hepatic enzymes in rats. Environ Sci Pollut Res Int 2015 4 28. 10.1007/s11356-015-4567-7 25913319

[45] Spitzweg C, Morris JC. The sodium iodide symporter: its pathophysiological and therapeutic implications. Clin Endocrinol (Oxf) 2002 11;57(5):559–74. 12390328 10.1046/j.1365-2265.2002.01640.x

[46] Rasmussen AK, Kayser L, Feldt-Rasmussen U, Bendtzen K. Influence of tumour necrosis factor-alpha, tumour necrosis factor-beta and interferon-gamma, separately and added together with interleukin-1 beta, on the function of cultured human thyroid cells. J Endocrinol 1994 11;143(2):359–65. 7829998 10.1677/joe.0.1430359

[47] Rasmussen AK, Diamant M, Blichert-Toft M, Bendtzen K, Feldt-Rasmussen U. The effects of interleukin-1beta (IL-1beta) on human thyrocyte functions are counteracted by the IL-1 receptor antagonist. Endocrinology 1997 5;138(5):2043–8. 9112403 10.1210/endo.138.5.5099

[48] Rasmussen AK, Kayser L, Bech K, Feldt-Rasmussen U, Perrild H, Bendtzen K. Differential effects of interleukin 1 alpha and 1 beta on cultured human and rat thyroid epithelial cells. Acta Endocrinol (Copenh) 1990 4;122(4):520–6. 2159205 10.1530/acta.0.1220520

[49] Berthois Y, Katzenellenbogen JA, Katzenellenbogen BS. Phenol red in tissue culture media is a weak estrogen: implications concerning the study of estrogen-responsive cells in culture. Proc Natl Acad Sci U S A 1986 4;83(8):2496–500. 3458212 10.1073/pnas.83.8.2496 PMC323325

[50] Rajoria S, Suriano R, George AL, Shanmugam A, Jussim C, Shin EJ, et al Estrogen activity as a preventive and therapeutic target in thyroid cancer. Biomed Pharmacother 2012 3;66(2):151–8. 10.1016/j.biopha.2011.11.010 22285105

[51] Frederiksen H, Jensen TK, Jorgensen N, Kyhl HB, Husby S, Skakkebaek NE, et al Human urinary excretion of non-persistent environmental chemicals: an overview of Danish data collected between 2006 and 2012. Reproduction 2014;147(4):555–65. 10.1530/REP-13-0522 24395915

[52] Frederiksen H, Kuiri-Hanninen T, Main KM, Dunkel L, Sankilampi U. A Longitudinal Study of Urinary Phthalate Excretion in 58 Full-Term and 67 Preterm Infants from Birth through 14 Months. Environ Health Perspect 2014 5 30. 10.1289/ehp.1307569 PMC4154216 24879654

[53] Hansen J, Boas M, Brorson M, Frederiksen H, Hartoft-Nielsen ML, Krogh-Rasmussen U, et al Technical report: Migration of phthalates on culture plates—an important challenge to consider for in vitro studies. Scandinavian Journal of Clinical and Laboratory Investigation. 10.3109/00365513.2015.1110857 26754760

[54] Rasmussen AK, Kayser L, Perrild H, Brandt M, Bech K, Feldt-Rasmussen U. Human thyroid epithelial cells cultured in monolayers. II. Influence of serum on thyroglobulin and cAMP production. Mol Cell Endocrinol 1996 2 5;116(2):173–9. 8647317 10.1016/0303-7207(95)03712-8

[55] Boas M, Feldt-Rasmussen U, Skakkebaek NE, Main KM. Environmental chemicals and thyroid function. Eur J Endocrinol 2006 5;154(5):599–611. 16645005 10.1530/eje.1.02128

