# Supplementary figures and images for: Phthalates Are Metabolised by Primary Thyroid Cell Cultures but Have Limited Influence on Selected Thyroid Cell Functions In Vitro
Source: PLoS One. 2016 Mar 17;11(3):e0151192. doi: 10.1371/journal.pone.0151192 (PMC4795645; doi:10.1371/journal.pone.0151192)

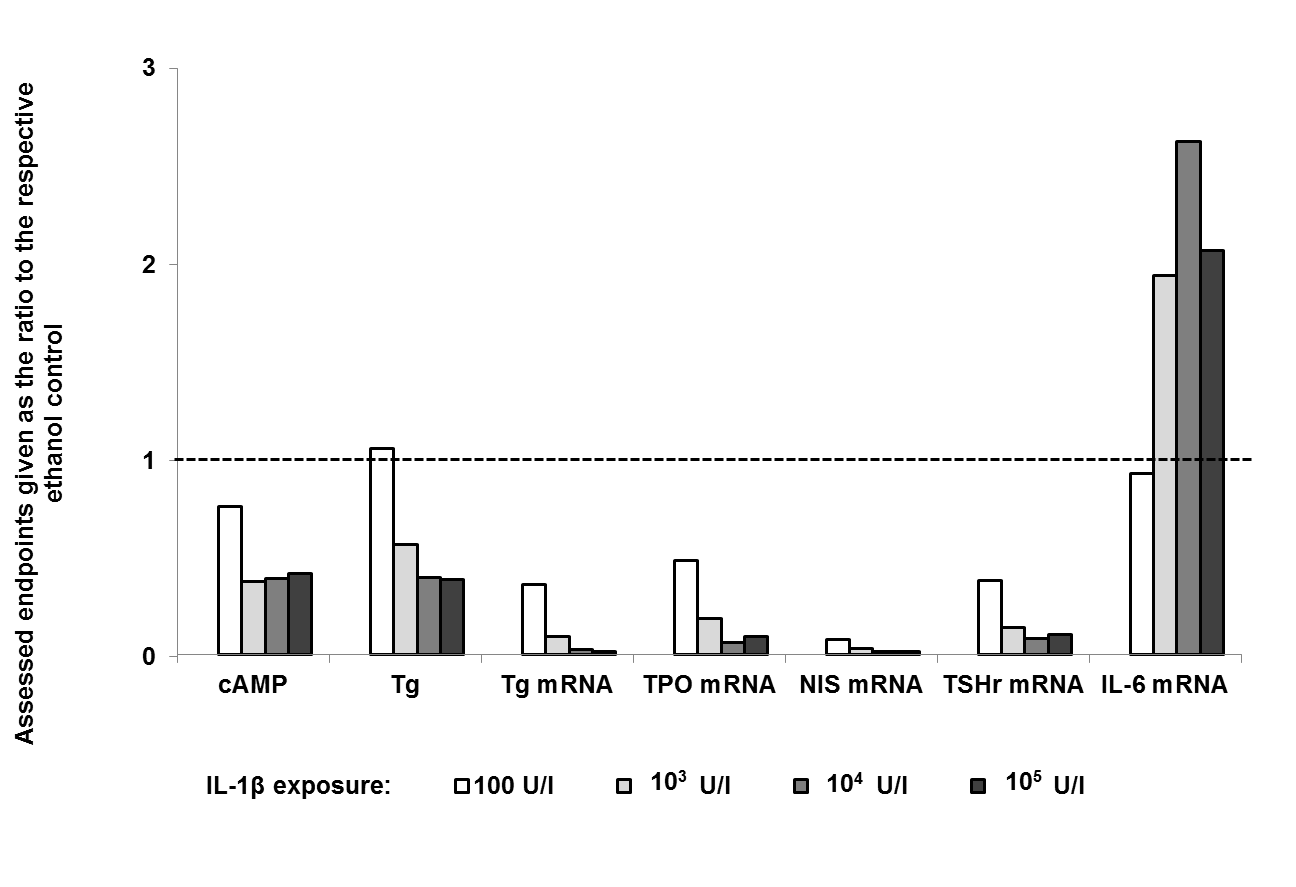

Supplement: S1 Fig — Cell cultures were exposed to IL-1β (100 to 105 U/l) for 72 hours (n = one culture in single determination). The dashed line indicates the level of the ethanol controls (ratio = 1). Tg- and cAMP-secretion was assessed in supernatants and mRNA in cells. cAMP: 3'-5'-cyclic adenosine monophosphate. Tg: thyroglobulin. TPO: thyroid peroxidase. NIS: sodium iodine symporter. TSHr: thyroid stimulating hormone receptor. IL: interleukin. (TIF) [file pone.0151192.s001.tif]

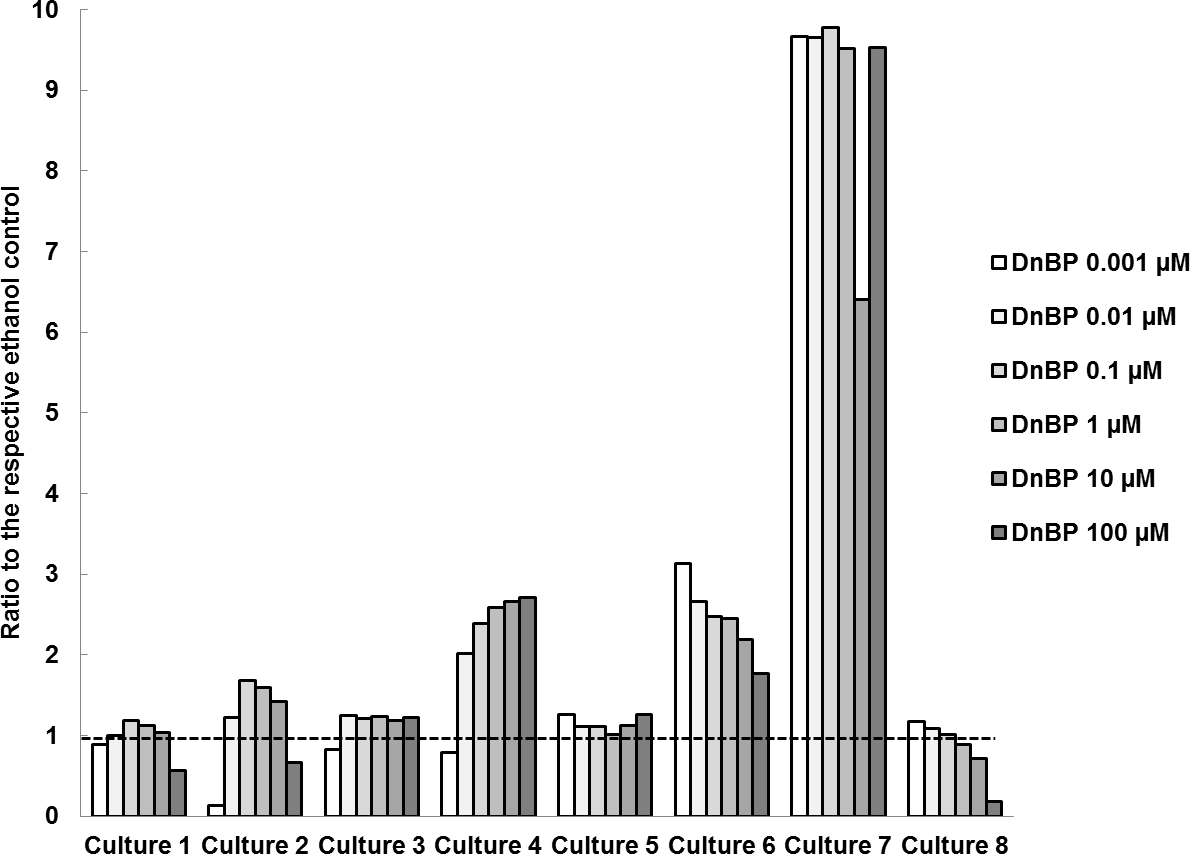

Supplement: S2 Fig — The dashed line indicates the level of the ethanol controls (ratio = 1). DnBP: di-n-butyl phthalate. (TIF) [file pone.0151192.s002.tif]

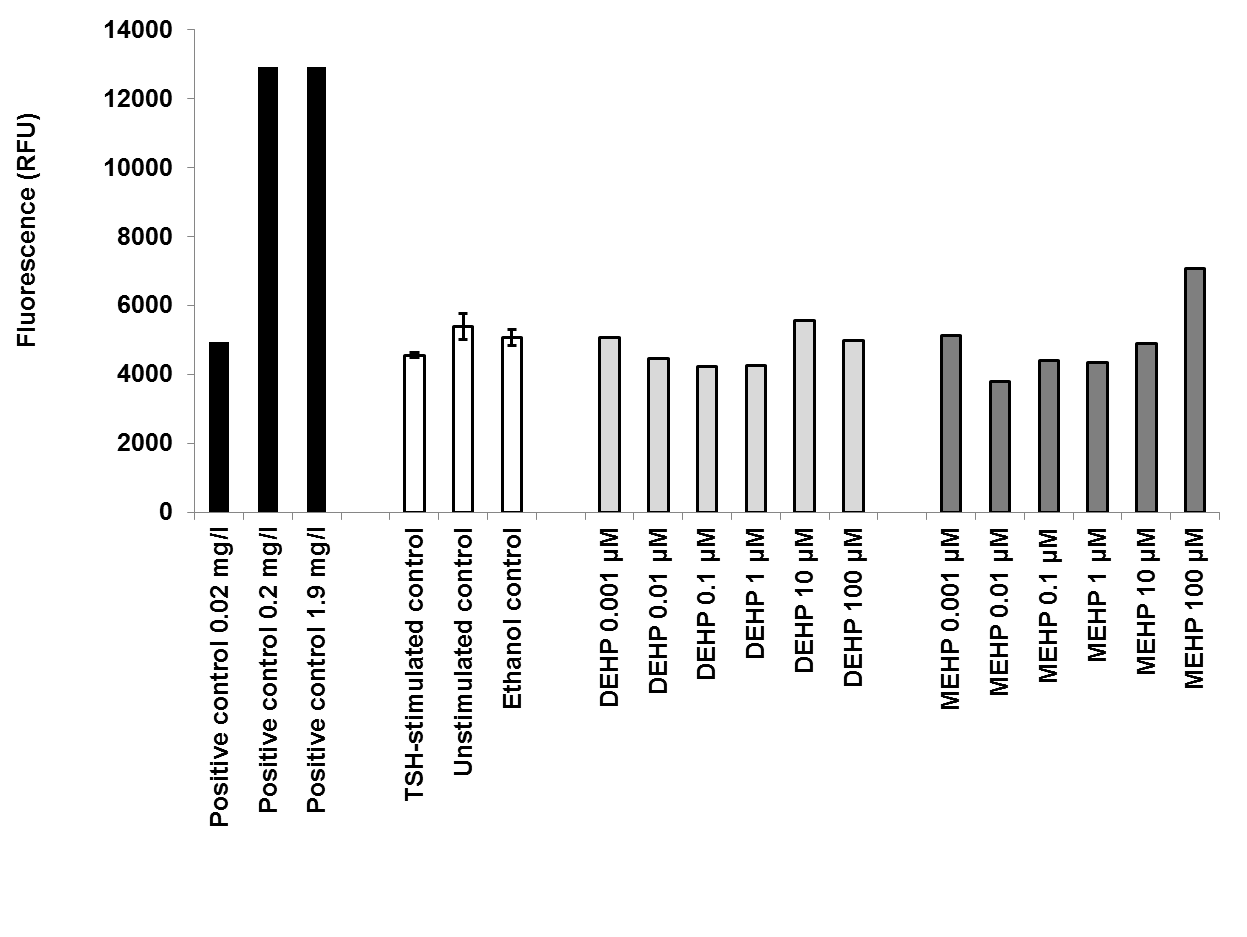

Supplement: S3 Fig — Cells were exposed to DEHP and MEHP, as well as positive cytotoxicity- (i.e. cell cultures exposed to Triton X-100) and negative culture- (i.e. cell cultures not exposed to phthalates) controls for 72 h. N = one culture in single determination except for the negative controls. The LDH-content was proportional to the produced fluorescence (given in relative fluorescence units (RFU)). DEHP: di-2-ethylhexyl phthalate. MEHP: mono-2-ethylhexyl phthalate. (TIF) [file pone.0151192.s003.tif]
