# Supplementary material for: Phthalates Are Metabolised by Primary Thyroid Cell Cultures but Have Limited Influence on Selected Thyroid Cell Functions In Vitro
Source: PLoS One. 2016 Mar 17;11(3):e0151192. doi: 10.1371/journal.pone.0151192 (PMC4795645; doi:10.1371/journal.pone.0151192)
Supplement: S1 Table — (PDF) [file pone.0151192.s004.pdf]

S1 Table. Sequences of the primers used in RT-qPCR.

Gene	Direction	Sequence
TG	forward	GGGCGGGCAGTCAGCAGAGAGTG
	reverse	CCATAGTGGGCAGCCTCGGGTGAG
TPO	forward	GGAGAGTGCTGGGATGGAAG
	reverse	GGATTTGCCTGTGTTTGGAA
SLC5A5 (NIS)	forward	ACCTTCTACACGGCTGTGGGCGGC
	reverse	CTCGGGTCAGGGTTAAAGTCCATG
TSHR	forward	GAATGCTTTTCAGGGACTATGCAAT
	reverse	ACAGCAGTGGCTTGGGTAAGAA
IL6	forward	AGAGTAACATGTGTGAAAGCAGCAA
	reverse	CCTCAAACTCCAAAAGACCAGTGA
B2M	forward	TGTGCTCGCGCTACTCTCTC
	reverse	CTGAATGCTCCACTTTTTCAATTCT
ACTB	forward	CTGGAACGGTGAAGGTGACA
	reverse	AAGGGACTTCCTGTAACAACGCA
GAPDH	forward	CATGAGAAGTATGACAACAGCCT
	reverse	AGTCCTTCCACGATACCAAAGT
ATP5B	forward	AACAATTTGCTCCCATTCAT
	reverse	CTCCAGCACCACCAAAAAGCCCAA
