# Supplementary material for: Phthalates Are Metabolised by Primary Thyroid Cell Cultures but Have Limited Influence on Selected Thyroid Cell Functions In Vitro
Source: PLoS One. 2016 Mar 17;11(3):e0151192. doi: 10.1371/journal.pone.0151192 (PMC4795645; doi:10.1371/journal.pone.0151192)
Supplement: S2 Table — Results of the outcome variables were analysed by paired T-test and experiment duration was 72 h. A: outcome from experiments with thyroid stimulation hormone (TSH) -starvation are compared to those without TSH-starvation. B: outcome from experiments without foetal bovine serum (FBS) are compared to those with FBS. Experiments were conducted in triplicates. cAMP: 3'-5'-cyclic adenosine monophosphate. IL: interleukin. NIS: sodium iodine symporter. Tg: thyroglobulin. TPO: thyroid peroxidase. TSHr: thyroid stimulating hormone receptor. (PDF) [file pone.0151192.s005.pdf]

S2 Table. The impact of TSH-starvation (A) and addition of FBS (B) during experiments.

**A.**	**Outcome variables**	**p-value**	**Transformation used**	**n**	**Mean of differences (95 %CI mean)**
Unstimulated cultures	cAMP	0.09	no	8	-
	Tg	0.1	no	7	-
	Tg mRNA	0.21	no	6	-
	TPO mRNA	0.28	no	6	-
	NIS mRNA	0.13	log10	6	-
	TSHr mRNA	0.15	no	6	-
	IL-6 mRNA	0.59	no	6	-
TSH-stimulated cultures	cAMP	0.03	no	8	0.60 (0.07;11.25)
	Tg	0.9	no	8	-
	Tg mRNA	0.11	no	6	-
	TPO mRNA	0.52	no	6	-
	NIS mRNA	0.83	no	6	-
	TSHr mRNA	0.80	no	6	-
	IL-6 mRNA	0.31	no	6	-
**B.**					
Unstimulated cultures	cAMP	0.42	no	3	-
	Tg	0.36	no	3	-
TSH-stimulated cultures	cAMP	0.04	no	3	0.93 (0.16;16.97)
	Tg	0.22	no	3	-

Footnote S2 Table: Results of the outcome variables were analysed by paired T-test and experiment duration was 72 h.
A: outcome from experiments with thyroid stimulation hormone (TSH) -starvation are compared to those without TSH-starvation.
B: outcome from experiments without foetal bovine serum (FBS) are compared to those with FBS. Experiments were conducted in triplicates. cAMP: 3'-5'-cyclic adenosine monophosphate. IL: interleukin. NIS: sodium iodine symporter. Tg: thyroglobulin. TPO: thyroid peroxidase. TSHr: thyroid stimulating hormone receptor.
