# Supplementary material for: Phthalates Are Metabolised by Primary Thyroid Cell Cultures but Have Limited Influence on Selected Thyroid Cell Functions In Vitro
Source: PLoS One. 2016 Mar 17;11(3):e0151192. doi: 10.1371/journal.pone.0151192 (PMC4795645; doi:10.1371/journal.pone.0151192)
Supplement: S3 Table — TSH-stimulated primary thyroid cells were cultured for 6, 24, 48 or 72 h, before supernatants and cells were harvested and outcome variables were analysed. All experiments were conducted in triplicates. cAMP: 3'-5'-cyclic adenosine monophosphate. IL: interleukin. NIS: sodium iodine symporter. Tg: thyroglobulin. TPO: thyroid peroxidase. TSHr: thyroid stimulating hormone receptor. (PDF) [file pone.0151192.s006.pdf]

S3 Table. ANOVA and Tukey post-hoc results from the study of the culture duration.

		2 way ANOVA		Tukey Post-hoc analysis		
**Outcome variables**	**n**	**p-value**	**Transformation used**	**Groups compared**	**P-value**	**Estimated difference in ratio (95% CI)**
**cAMP**	7	<0.0001	log10	6 to 24 h	<0.0001	0.19 (0.12;0.32)
				6 to 48 h	<0.0001	0.06 (0.04;0.10)
				6 to 72 h	<0.0001	0.04 (0.03;0.07)
				24 to 48 h	<0.0001	0.33 (0.20;0.54)
				24 to 72 h	<0.0001	0.22 (0.13;0.37)
				48 to 72 h	0.17	0.68 (0.41;0.89)
**Tg**	7	<0.0001	log10	6 to 24 h	<0.0001	0.30 (0.21;0.43)
				6 to 48 h	<0.0001	0.11 (0.08;0.16)
				6 to 72 h	<0.0001	0.06 (0.04;0.08)
				24 to 48 h	<0.0001	0.37 (0.25;0.53)
				24 to 72 h	<0.0001	0.19 (0.13;0.27)
				48 to 72 h	0.0004	0.51 (0.35;0.74)
**Tg mRNA**	7	<0.0001	log10	6 to 24 h	<0.0001	0.38 (0.26;0.55)
				6 to 48 h	<0.0001	0.24 (0.17;0.36)
				6 to 72 h	<0.0001	0.26 (0.18;0.39)
				24 to 48 h	0.02	0.65 (0.44;0.95)
				24 to 72 h	0.08	-
				48 to 72 h	0.92	-
**TPO mRNA**	6	<0.0001	log10	6 to 24 h	0.0001	0.47 (0.32;0.67)
				6 to 48 h	<0.0001	0.34 (0.23;0.49)
				6 to 72 h	<0.0001	0.32 (0.22; 0.47)
				24 to 48 h	0.10	-
				24 to 72 h	0.048	0.69 (0.48; 0.997)
				48 to 72 h	0.98	-
**NIS mRNA**	6	<0.001	log10	6 to 24 h	<0.0001	0.05 (0.02;0.11)
				6 to 48 h	<0.0001	0.05 (0.02;0.10)
				6 to 72 h	<0.0001	0.07 (0.03;0.15)
				24 to 48 h	0.99	-
				24 to 72 h	0.70	-
				48 to 72 h	0.53	-
**TSHr mRNA**	7	<0.0001	log10	6 to 24 h	<0.0001	0.54 (0.41;0.71)
				6 to 48 h	<0.0001	0.45 (0.34;0.59)
				6 to 72 h	<0.0001	0.46 (0.35;0.61)
				24 to 48 h	0.26	-
				24 to 72 h	0.43	-
				48 to 72 h	0.99	-
**IL-6 mRNA**	7	0.02	log10	6 to 24 h	0.03	0.46 (0.23;0.93)
				6 to 48 h	0.03	0.47 (0.23;0.95)
				6 to 72 h	0.06	-
				24 to 48 h	1.0	-
				24 to 72 h	0.99	-
				48 to 72 h	0.99	-

Footnote to S3 Table: TSH-stimulated primary thyroid cells were cultured for 6, 24, 48 or 72 h, before supernatants and cells were harvested and outcome variables were analysed. All experiments were conducted in triplicates. cAMP: 3'-5'-cyclic adenosine monophosphate.
IL: interleukin. NIS: sodium iodine symporter. Tg: thyroglobulin. TPO: thyroid peroxidase. TSHr: thyroid stimulating hormone receptor.
