# Supplementary material for: Phthalates Are Metabolised by Primary Thyroid Cell Cultures but Have Limited Influence on Selected Thyroid Cell Functions In Vitro
Source: PLoS One. 2016 Mar 17;11(3):e0151192. doi: 10.1371/journal.pone.0151192 (PMC4795645; doi:10.1371/journal.pone.0151192)
Supplement: S4 Table — If log10 of data was used, the estimated differences between groups are expressed as ratios, e.g. the mean cAMP-secretion from 10 μM DEHP-exposed cells was 21% lower than the mean cAMP-secretion from 0.001 μM DEHP-exposed cells, and lies with 95% certainty between 37% below and 0% below the mean of 0.001 μM DEHP-exposed cells. The hyphen (-) indicates that no post-hoc analysis was made due to insignificant 2 way ANOVA results. cAMP: 3'-5'-cyclic adenosine monophosphate. DEHP: di-2-ethylhexyl phthalate. DEP: di-ethyl phthalate. DnBP: di-n-butyl phthalate. MEHP: mono-2-ethylhexyl phthalate. MnBP: mono-n-butyl phthalate. IL: interleukin. NIS: sodium iodine symporter. Tg: thyroglobulin. TPO: thyroidperoxidase. TSH: thyroid stimulating hormone. TSHr: thyroid stimulating hormone receptor. (PDF) [file pone.0151192.s007.pdf]

S6 Table. Overview of 2 way ANOVA and post-hoc Tukey results from phthalate-exposed (72 h) thyroid cell cultures, in TSH- and unstimulated controls.

Outcome variables	Phthalate	+/- TSH	n (S,D or T)	p-value ANOVA	Transformed data used	p-value Tukey	Groups compared	Estimated ratio†/difference (95% CI) Tukey
cAMP	DEP	+	6	0.10	no	-	-	-
	DnBP	+	8	0.10	log10	-	-	-
	MnBP	+	6	0.13	log10	-	-	-
	DEHP	+	9	0.0006	log10	0.05	10 versus 0.001 µM	0.79 (0.63; 1.00)
						0.005	10 versus 0.01 µM	0.75 (0.59; 0.94)
						0.008	10 versus 0.1 µM	0.75 (0.60; 0.95)
						0.01	10 versus 1 µM	0.76 (0.60; 0.96)
						0.80	10 versus 100 µM	0.90 (0.71; 1.13)
						0.003	10 µM versus control	0.73 (0.58; 0.92)
	MEHP	+	3	0.01	log10	0.04	100 versus 0.001 µM	0.12 (0.02; 0.91)
						0.03	100versus 0.01 µM	0.11 (0.02; 0.85)
						0.02	100 versus 0.1 µM	0.09 (0.01; 0.67)
						0.02	100 versus 1 µM	0.10 (0.01; 0.71)
						0.04	100 versus 10 µM	0.12 (0.02; 0.89)
						0.01	100 µM versus control	0.09 (0.01; 0.65)
	DEP	-	5	0.95	no	-	-	-
	DnBP	-	5	0.55	no	-	-	-
	MnBP	-	3	0.88	no	-	-	-
Tg	DEP	+	11	0.49	log10	-	-	-
	DnBP	+	10	0.10	log10	-	-	-
	MnBP	+	10	0.11	log10	-	-	-
	DEHP	+	9	0.76	log10	-	-	-
	MEHP	+	3	0.002	log10	0.008	100 versus 0.001 µM	0.53 (0.33;0.86)
						0.002	100 versus 0.01 µM	0.47 (0.29;0.76)
						0.003	100 versus 0.1 µM	0.49 (0.31;0.80)
						0.003	100 versus 1 µM	0.48 (0.30;0.78)
						0.02	100 versus 10 µM	0.56 (0.35;0.90)
						0.01	100 µM versus contro	0.55 (0.34;0.89)
	DEP	-	8	0.75	log10	-	-	-
	DBP	-	6	0.58	log10	-	-	-
	MBP	-	6	0.54	log10	-	-	-
Tg mRNA	DEP	+	2	0.43	log10	-	-	-
	DnBP	+	2	0.06	log10	-	-	-
	DEHP	+	7	0.92	log10	-	-	-
	DEP	-	3	0.42	no	-	-	-
TPO mRNA	DEP	+	2	0.29	log10	-	-	-
	DnBP	+	3	0.47	log10	-	-	-
	DEHP	+	7	0.47	log10	-	-	-
	DEP	-	3	0.29	log10	-	-	-
NIS mRNA	DEP	+	2	0.58	no	-	-	-
	DnBP	+	3	0.28	log10	-	-	-
	DEHP	+	7	0.59	log10	-	-	-
	DEP	-	3	0.30	log10	-	-	-
TSHR mRNA	DEP	+	2	0.30	log10	-	-	-
	DnBP	+	3	0.55	no	-	-	-
	DEHP	+	7	0.76	log10	-	-	-
	DEP	-	3	0.13	log10	-	-	-
IL-6 mRNA	DEP	+	2	0.41	no	-	-	-
	DnBP	+	3	0.37	log10	-	-	-
	DEHP	+	6	0.80	log10	-	-	-
	DEP	-	3	0.75	no	-	-	-

Footnote to S6 Table: If log10 of data was used, the estimated differences between groups are expressed as ratios, e.g. the mean cAMP-secretion from 10 µM DEHP-exposed cells was 21% lower than the mean cAMP-secretion from 0.001 µM DEHP-exposed cells, and lies with 95% certainty between 37% below and 0% below the mean of 0.001 µM DEHP-exposed cells. The hyphen (-) indicates that no post-hoc analysis was made due to insignificant 2 way ANOVA results. cAMP: 3'-5'-cyclic adenosine monophosphate. DEHP: di-2-ethylhexyl phthalate. DEP: di-ethyl phthalate. DnBP: di-n-butyl phthalate. MEHP: mono-2-ethylhexyl phthalate. MnBP: mono-n-butyl phthalate. IL: interleukin. NIS: sodium iodine symporter. Tg: thyroglobulin. TPO: thyroidperoxidase. TSH: thyroid stimulating hormone. TSHr: thyroid stimulating hormone receptor.
